# Villous Adenoma Arising in the Urethra of a Female with Bladder Augmentation History: A Case Report and Review of the Literature

**DOI:** 10.5146/tjpath.2020.01502

**Published:** 2021-05-15

**Authors:** Hale Demir, Selçuk Cin, Sinharib Citgez, Nesrin Uygun

**Affiliations:** Department of Pathology, Amasya University, School of Medicine, Amasya, Turkey; Bagcilar Training and Research Hospital, Istanbul, Turkey; Department of Urology, Istanbul University-Cerrahpasa, Cerrahpasa School of Medicine, Istanbul, Turkey; Department of Pathology, Istanbul University-Cerrahpasa, Cerrahpasa School of Medicine, Istanbul, Turkey

**Keywords:** Villous adenoma, Urethra, Urinary tract, Bladder augmentation

## Abstract

Villous adenomas (VAs) in the female urethra are rare with only seven cases in the English literature to our knowledge. In patients with bladder augmentation cystoplasty, the neoplasia development risk increases and most of these develop in the neobladder or anastomosis line. Only two cases of VA developing from the native bladder mucosa have been reported. Physical examination of a 76-year-old female who had a history of augmentation cystoplasty revealed a caruncula-like structure protruding from the urethral meatus. The urinary USG showed that the lesion had no relation with the bladder. The lesion was excised. Microscopically, it consisted of villous structures covered with pseudostratified intestinal type epithelium. Low-grade dysplasia was present in the epithelium but high-grade dysplasia or in-situ/invasive carcinoma was not observed. Immunohistochemical study showed positivity for CK7, CK20, EMA, CEA and CDX2. The case was reported as VA of the urethra. We presented the first VA case arising in the urethra of a female patient with intestinal bladder augmentation. Excision is curative for pure VAs. Transformation to carcinoma or recurrence has not been reported. However, in one third of the cases, a malignant tumor may accompany the lesion. Therefore, all excision material should be examined carefully. Routine endoscopic follow-up should be performed in cases with bladder augmentation.

## INTRODUCTION

Villous adenomas (VAs) of the urinary tract are rare with only two case series and around 20 scattered case reports in the literature (1–3). Histologically and immunohistochemically, these tumors are similar to VAs of the gastrointestinal system (1,3). They are frequently seen in elderly patients with a predilection for the urachus, dome and trigone of the bladder (1,4). Male predominance has been reported (1,2,5). VAs of the female urethra are very rare with only seven cases in the English literature to our knowledge (Table 1) (1,6–11).

**Table 1:** Summary of our case and literature review of cases of villous adenoma arising in the female urethra.

**Reference**	**Age** **(years)**	**Symptoms**	**Size** **(mm)**	**Coexisting** **malignancy**	**Treatment**	**Follow-up** **(months)**	**Outcome**
Powell et al. (8)	59	Polypoid lesion protruding from the urethral meatus, hematuria	8x8	Adenocarcinoma	Transurethral excision	15	Alive
Howells and Baylis (10)	70	Vaginal discharge, dysuria	30x20x15	Absent	Surgical resection	22	Alive
Raju et al. (9)	58	Asymptomatic mass increasing in size	10	Absent	Tumor excision	72	Alive with no recurrence
Morgan et al. (11)	87	Asymptomatic polypoid urethral mass	20x15x10	Absent	Tumor excision	24	Dead*
Cheng et al. (1)	81	Hematuria	Unknown	Absent	Unknown	Unknown	Unknown
Noel et al. (7)	49	Painful paraurethral tumor increasing in size, hematuria	30x28x25	Adenosquamous carcinoma	Wide tumor excision	16	Alive with no recurrence
Qin et al. (6)	63	A mass in the urethral orifice increasing in size	40x30	Well differentiated adenocarcinoma	Whole urethra and part of the bladder resection	11	Alive with no recurrence
Present case	76	Polypoid lesion protruding from the urethral meatus	45x20x15	Absent	Tumor excision	28	Alive with no recurrence

* The patient died 2 years later of a cerebral infarction.

In the etiology, it has been considered that urinary tract VAs might develop from the cloacal remnants from which the distal colorectum, bladder and urethra originate during embryogenesis. On the other hand, they may also be a product of the chronic irritation-metaplasia-dysplasia-carcinoma sequence (3).

In patients with bladder augmentation cystoplasty, it is reported that the risk of carcinoma development increases (4). Most of these carcinomas develop in the neobladder or anastomosis line. Only 2 cases of VA that developed from the native bladder mucosa have been reported (Table 2) (4,12). It is thought that the development of VA in patients with bladder augmentation supports the second theory.

**Table 2:** Villous adenoma arising from the urinary mucosa in the patients with a history of augmentation.

**Reference**	Age *	**Sex**	**Original disease**	**Segment for ECP**	**Duration from ECP (yrs.)**	**Tumor site**	**Coexisting** **malignancy**	**Treatment**	**Follow-up**
Lin et al. (12)	25	F	Small capacity- neurogenic bladder	Stomach	16	Native bladder	Adenocarcinoma	Resection of recontracted stomach part and wedge excision of tumor	Active surveillance without adjuvant therapy
Nayak et al. (4)	52	M	Posterior urethral valves, ileal diversion at the age of 6 months	Colon	32	Native bladder, then bilateral pelvis and ureters	Adenocarcinoma and Neuroendocrine carcinoma	TUR, then bilateral nephrectomy, ureterectomy, cystoprostatectomy	Recurrence in urinary tract (+), distant metastasis (+), alive after 3 months
Present case	76	F	Hypersensitive bladder with small capacity	Ileum	30	Urethra	Absent	Tumor excision	No recurrence after 28 months

* Age at villous adenoma diagnosis, ECP: Enterocystoplasty.

We present the first VA case arising in the urethra of a female patient with intestinal bladder augmentation.

## CASE REPORT

A 76-year-old female patient was hospitalized because of high urea and creatinine levels. She had a history of intestinal augmentation cystoplasty due to small capacity hypersensitive bladder 30 years ago. The ileal segment was augmented to the bladder dome. The bladder trigone and urethra were not interfered with. Clean intermittent catheterization (CIC) was recommended to the patient after the first augmentation, but she did not do it regularly.

She was diagnosed as postrenal acute renal failure. A catheter was inserted and the urea-creatinine levels began to decrease. She had bilateral hydroureteronephrosis. After 4 months, physical examination revealed a caruncula-like structure protruding from the urethral meatus. The urinary USG revealed that there was no relation with the bladder. The urethral lesion was excised, and then the bilateral hydroureteronephrosis regressed. CIC was not recommended for postoperative follow-up, as the patient had no residual urine.

The macroscopic examination of the lesion revealed a cream-white colored, fragile and polypoid mass, 4.5x2x1.5 cm in size. Its base was 1.5x1 cm in size, hemorrhagic and brown colored. On the cut sections, it was composed of thin papillary structures adhering to a fibrous core.

**Figure 1:**
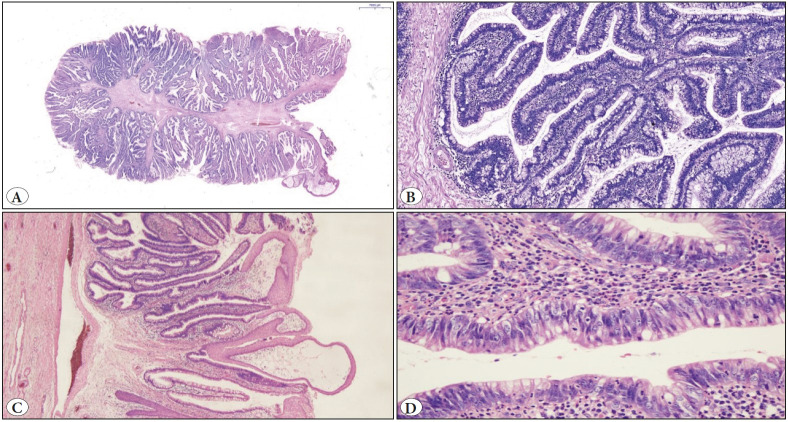
Microscopic view of the urethral villous adenoma. **A)** A slide photograph shows thin papillary structures adhering to a fibrous core (H&E; x4). **B)** Villous structures covered with pseudostratified intestinal type epithelium (H&E; x100). **C)** Squamous metaplasia areas (H&E; x40). **D)** Low-grade dysplasia of adenomatous epithelium (H&E; x400).

**Figure 2:**
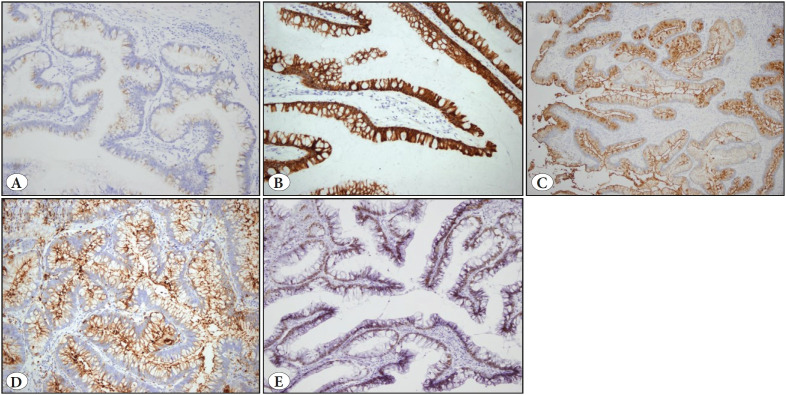
Immunohistochemical panel. Positivity for **A)** CK7 (IHC; x200), **B)** CK20 (IHC; x200), **C)** EMA (IHC; x200), **D)** CEA (IHC; x200), **E)** CDX2 (IHC; x200).

On microscopic examination, the tumor consisted of villous structures covered with pseudostratified intestinal type epithelium. Low-grade dysplasia and occasional squamous metaplasia areas were present in the epithelium (Figure 1). All material was investigated and high-grade dysplasia or in-situ / invasive carcinoma was not observed. Immunohistochemical study showed positivity for CK7, CK20, EMA, CEA and CDX2 (Figure 2).

The case was reported as VA of the urethra. The adenomatous epithelium was adjacent to the surgical margin. However, there was no surgical recurrence in the 28-month follow-up. During follow-up, there was no renal dysfunction or hydronephrosis. The patient stated that she was more comfortable after the operation and could urinate.

## DISCUSSION

Urinary tract VAs are benign glandular lesions which are histologically and immunohistochemically similar to VAs of the gastrointestinal tract (1,3,13). They are mostly seen in patients aged 33 to 79 years with a mean age of 57 years (14). Male predominance has been reported (1,2,5). Patients usually present with hematuria, irritation symptoms and rarely mucusuria (1,15). Back or flank pain, fever, abdominal discomfort and weight loss can be seen in patients with pelvic VAs (16). There is no specific diagnostic finding on the USG, CT, MRI or cystoscopy (17,18).

In the past, various terms have been used to describe these tumors as villous adenoma, tubulovillous adenoma, villous metaplasia of the intestinal type with dysplasia, and enteric adenoma. However, using the villous tumor or villous polyp terms must be avoided because they can lead to confusion with prostatic-type polyps (19).

Urinary tract VAs mostly arise in the urachus, dome, or trigone of the bladder (1,4). Cases located in the urethra, renal pelvis, and ureter have also been reported (6,20–22). Dong et al. have reported that they found only 8 cases of pelvic VA in the English literature and presented 2 additional cases (22). Qin et al. also reported that they found only 11 cases with urethral VA between 1981 and 2003. Six of these, and the case which they presented, were female (6). Our presented case is therefore the eighth VA case arising in the female urethra, as far as we are aware.

Two possibilities have been reported in the etiology of VAs. According to the first theory, the distal colorectum, bladder and urethra originate from the cloacal tissue during embryogenesis, and these tumors may develop from the cloacal remnants. This also explains the morphological and cytogenetic similarity of the urinary tract lesions and their counterparts in the gastrointestinal system. According to the second theory, VAs are a product of the irritation-metaplasia-dysplasia-carcinoma sequence (3). Intestinal metaplasia of the urinary tract is often associated with chronic inflammation, in particular infection, stones and chemical injury (16). The presence of neutral mucins, acidic sulfomucins, and sialomucins in both cystitis glandularis and VA, and the similar genetic characteristics in the dysplastic regions of the metaplastic mucosa and VA support this theory (3,23).

Augmentation cystoplasty using the colon, ileum or stomach is an accepted reconstructive option for patients with intractable incontinence and poor bladder compliance due to neurogenic and non-neurogenic disorders (6). However, it has been reported that the risk of developing bladder carcinoma increases 7-8 times with ileum and colon segments, and 14-15 times with gastric segments (4,12). The most common histologic types are adenocarcinoma and urothelial carcinoma (12). The incidence of VAs after cystoplasty is very low and most of them develop in the neobladder or anastomosis line (24). To our knowledge, only 2 cases of VAs originating from the native bladder mucosa have been reported (4,12). It is thought that the development of bladder VA in patients with a history of augmentation supports the irritation-metaplasia-dysplasia-carcinoma sequence theory (4,12).

Our presented case had a history of intestinal augmentation cystoplasty 30 years ago. However, VA had developed in the urethral mucosa and protruded from the urethral meatus. USG revealed that there was no relation with the bladder. Our case is the first VA in a female urethra with a bladder augmentation history. This case could also be another example supporting the second theory.

Histologically, VAs consist of long villoglandular structures with a central fibrovascular core and these structures are lined by pseudostratified columnar epithelium (1,13). The epithelial cells display nuclear stratification, crowding, hyperchromasia and occasional prominent nucleoli. Variable mitotic figures are seen in situ and in the invasive component (1). There is frequently a background of chronic cystitis and association with intestinal/squamous metaplasia, cystitis cystica and cystitis glandularis (2).

Two-thirds of the cases may have simultaneous malignant tumors such as urothelial carcinoma, and in situ or infiltrative adenocarcinoma (2). Therefore, all of the excision material should be examined carefully (2). In our case, we investigated all excision material and there was no high-grade dysplasia or in situ/invasive carcinoma focus.

Immunohistochemically, CK20 positivity was reported at a rate of 100%, while CEA and EMA can be positive. CDX2 positivity is also reported (25). CK7 positivity is observed in approximately half of the cases (1,3,26). Our case showed positivity for all these markers.

Distinction from tumor metastasis of adjacent organs such as colon, the female genital system, and the prostate is important. For females with genital system adenocarcinoma, Ca125, ER, PR can be helpful as diagnostic markers (6). Morphological differential diagnosis between metastatic adenocarcinoma of the gastrointestinal system and urinary tract VAs is impossible. CK7 positivity can support VAs of the urinary tract (3,6). In the male urethra, morphological features of prostatic ductal adenocarcinoma can easily mimic VA (27). Expression of prostatic lineage markers such as PSA and PSMA could be helpful to differentiate the two entities (13).

Cytologically, it is difficult to distinguish VAs from other glandular lesions. However, VAs must be considered in the differential diagnosis when many glandular cells or mucinous cells without atypia are recognized in the urine (28).

Excision is curative for pure VAs. Carcinoma transformation and recurrence were not reported in pure cases during a mean follow up of 9.9 years (1). However, more aggressive treatment may be indicated for the VAs coexisting with a malignant tumor as adenocarcinoma to prevent recurrence and metastasis (1,6). In VAs of yjr calyx, the adenoma may result in atypical hyperplasia and cancerization due to continuous inflammation stimulation, and surgical resection is therefore recommended (22). In the ureters, tumor growth with mucus retention may be the main cause of ureteral obstruction, and total excision of the tumor prevents early obstruction (29). In patients with bladder augmentation, long-term active surveillance is necessary in terms of neoplasia development (12,24). In our presented case, the urethral tumor was excised. Although the surgical margin was microscopically positive, there was no recurrence in 28-month follow-up.

In conclusion, our case is the first VA arising in the female urethra with a history of bladder augmentation. The present case report supports the irritation-metaplasia-dysplasia-carcinoma sequence theory. Excision is curative for pure VAs. Transformation to carcinoma or recurrence has not been reported. However, there may be an accompanying malignant tumor such as adenocarcinoma, urothelial carcinoma etc. in one-third of the cases. Therefore, all excision material should be examined carefully. Routine endoscopic follow-up should be performed in cases with bladder augmentation. Further studies are needed to establish the nature of urinary tract VAs.

## Conflict of INTEREST

The authors declare no conflict of interest.

## References

[ref-1] Cheng L., Montironi R., Bostwick D. G. (1999). Villous adenoma of the urinary tract: a report of 23 cases, including 8 with coexistent adenocarcinoma. Am J Surg Pathol.

[ref-2] Seibel Jeffrey L., Prasad Saket, Weiss Robert E., Bancila Edita, Epstein Jonathan I. (2002). Villous adenoma of the urinary tract: a lesion frequently associated with malignancy. Hum Pathol.

[ref-3] Fernandes Gwendolyn, Munde Shital, Rojekar Amey (2017). Pure Villous Adenoma of the Vesicoureteric Junction Presenting as Pyonephrosis. J Clin Diagn Res.

[ref-4] Nayak Anupma, Depasquale Brittany, Vergara Norge, Guzzo Thomas A., Lal Priti (2019). Villous Adenoma Arising in the Native Bladder Mucosa and the Upper Urinary Tract With Coexisting Neuroendocrine Carcinoma Following Augmentation Cystoplasty. Int J Surg Pathol.

[ref-5] Atik Esin, Akansu Bülent, Davarci Mürsel, Inci Mehmet, Yalcinkaya Fatih, Rifaioglu Murat (2012). Villous adenoma of the urinary bladder: rare location. Contemp Oncol (Pozn).

[ref-6] Qin Lu-Feng, Liang Ye, Xing Xiao-Ming, Wu Hui, Yang Xue-Cheng, Niu Hai-Tao (2019). Villous adenoma coexistent with focal well-differentiated adenocarcinoma of female urethral orifice: A case report and review of literature. World J Clin Cases.

[ref-7] Noel Jean-Christophe, Fayt Isabelle, Aguilar Sergio Fernandez (2006). Adenosquamous carcinoma arising in villous adenoma from female vulvar urethra. Acta Obstet Gynecol Scand.

[ref-8] Powell I., Cartwright H., Jano F. (1981). Villous adenoma and adenocarcinoma of female urethra. Urology.

[ref-9] Raju G. C., Roopnarinesingh A., Woo J. (1987). Villous adenoma of female urethra. Urology.

[ref-10] Howells M. R., Baylis M. S. (1985). Benign urethral villous adenoma. Case report. Br J Obstet Gynaecol.

[ref-11] Morgan D. R., Dixon M. F., Harnden P. (1998). Villous adenoma of urethra associated with tubulovillous adenoma and adenocarcinoma of rectum. Histopathology.

[ref-12] Lin Ting-Po, Chen Marcelo, Hsu Jong-Ming, Sheu J. C. (2014). Adenocarcinoma arising from tubulovillous adenoma in a native bladder following gastrocystoplasty. Pediatr Surg Int.

[ref-13] McKenney Jesse K. (2019). Precursor lesions of the urinary bladder. Histopathology.

[ref-14] Sung Wooseuk, Park Byung-Dae, Lee Sun, Chang Sung-Goo (2008). Villous adenoma of the urinary bladder. Int J Urol.

[ref-15] Karnjanawanichkul Watid, Tanthanuch Monthira, Mitarnun Winyou, Pripatnanont Choosak (2013). Renal pelvic villous adenoma presented with mucusuria: report of a case and literature review. Int J Urol.

[ref-16] Hudson Jill, Arnason Thomas, Merrimen Jennifer L. O., Lawen Joseph (2013). Intestinal type villous adenoma of the renal pelvis. Can Urol Assoc J.

[ref-17] Pal Dilip Kumar (2015). Villous adenoma of the urinary bladder. J Cancer Res Ther.

[ref-18] Kato Yoichiro, Konari Susumu, Obara Wataru, Sugai Tamotsu, Fujioka Tomoaki (2013). Concurrence of villous adenoma and non-muscle invasive bladder cancer arising in the bladder: a case report and review of the literature. BMC Urol.

[ref-19] Tamboli P., Ro J. Y. (2000). Villous adenoma of urinary tract: a common tumor in an uncommon location. Adv Anat Pathol.

[ref-20] Fernando Val-Bernal J., Torío B., Mayorga M., García-Arranz P., Garijo M. F. (2001). Concurrent tubulovillous adenoma and transitional cell carcinoma associated with diffuse gastric and intestinal metaplasia of the defunctioned ureter. Pathol Res Pract.

[ref-21] Bhat Suresh, Chandran Venu (2010). Villous adenoma of the renal pelvis and ureter. Indian J Urol.

[ref-22] Dong Chunge, Yang Youping, Wu Siying, Chen Guorong (2015). Clinicopathological analysis of two cases with pelvis villous adenoma and review of relevant literature. J Cancer Res Ther.

[ref-23] Channer J. L., Williams J. L., Henry L. (1993). Villous adenoma of the bladder. J Clin Pathol.

[ref-24] Hayashi Yutaka, Shiyanagi Satoko, Nagae Itsuro, Ishizaki Tetsuo, Kasuya Kazuhiko, Katsumata Kenji, Yamataka Atsuyuki, Tsuchida Akihiko (2016). A case of tubular adenoma developing after bladder augmentation: Case report and literature review. Int J Surg Case Rep.

[ref-25] Wang Jindong, Manucha Varsha (2016). Villous Adenoma of the Urinary Bladder: A Brief Review of the Literature. Arch Pathol Lab Med.

[ref-26] Nakamura Yasuhiro, Orikasa Kazuhiko, Fujishima Fumiyoshi, Shibahara Yukiko, Saito Ryoko, Ohkubo Teppei, Ueno Seiji, Sasano Hironobu (2011). A case of villous adenoma of the urinary bladder with tubulovillous architecture: characterization by immunohistochemical analysis. Pol J Pathol.

[ref-27] Sato Katsuaki, Tachibana Hironori, Tsuzuki Toyonori, Ueda Yoshimichi, Katsuda Shogo (2006). Prostatic ductal adenocarcinoma mimicking villous adenoma of the urethra. Virchows Arch.

[ref-28] Ishikawa Ryou, Kadota Kyuichi, Hayashi Toshitetsu, Motoyama Mutsumi, Matsunaga Toru, Miyai Yumi, Katsuki Naomi, Kushida Yoshio, Haba Reiji (2016). Cytopathological features of villous adenoma of the urinary bladder in urine: A rare case report. Diagn Cytopathol.

[ref-29] Shih Chi-Min, Wu Sheng-Chuan, Lee Chueng-Chen, Pan Chin-Chen (2007). Villous adenoma of the ureter with manifestation of mucus hydroureteronephrosis. J Chin Med Assoc.

